# Refining Risk Estimates in Hereditary Nonpolyposis Colorectal Cancer: Are We There Yet?

**DOI:** 10.1093/jncics/pkaa030

**Published:** 2020-04-27

**Authors:** Patrick M Lynch, Mala Pande

**Affiliations:** Department of Gastroenterology, Hepatology and Nutrition, The University of Texas MD Anderson Cancer Center, Houston, TX, USA

The most important benefit to be derived from detecting the presence of a pathogenic variant in cancer susceptibility genes lies in the ability to perform predictive testing in at-risk relatives and to then undertake risk-reducing interventions. In the case of the mismatch repair (MMR) genes that predispose to hereditary nonpolyposis colorectal cancer, or Lynch syndrome, the recognition of carrier status enables targeted, aggressive surveillance, generally by means of colonoscopy, the intent of which is to reduce colorectal cancer risk by means of eliminating its precursor, the adenoma.

Of the MMR genes—*MLH1*, *MSH2*, *MSH6*, *PMS2*, (and *EPCAM,* which when mutated inactivates the downstream MSH2)—the first two to be identified, *MSH2* and *MLH1*, were detected through the study of very highly penetrant families with striking aggregations of colorectal cancer, with onset as early as age 40, 30, or even 20 years. As the other genes were found, it soon became evident that cancer risks were likely lower in patients having pathogenic variants in them. However, the emergence of clinical practice guidelines has generally treated risks, and thus the need for surveillance, as similar for patients irrespective of the gene mutated. Reasons for this have likely included concerns that data were too limited to allow stratification of risk on a gene-by-gene basis, such that tailoring of surveillance would be considered premature and cavalier.

In this issue of the *JNCI Cancer Spectrum* Wang et al. (1) present a meta-analysis of colorectal cancer risks, using aggregated figures from the most well-sourced data repositories. The 10 studies included in the meta-analysis were notably heterogeneous for a variety of reasons, including study design (largely retrospective, with only one prospective cohort), focused exclusively on founder mutations (3 studies) and ascertainment methods (clinic vs population-based). By performing systematic leave-1-study-out sensitivity analysis, the authors established that risk estimates for *MLH1* and *MSH2* were robust to the removal of studies. Risk estimates for *MSH6* were less stable to the removal of studies given that they were based on fewer studies.

An important modifier of colorectal cancer risk is guideline-recommended colonoscopic surveillance or prophylactic surgery. The authors provide a subanalysis of risk estimates, both for studies that focus on unscreened populations and for those presumed to be a mix of screened and unscreened populations. Overall, there was considerable overlap in the 95% confidence intervals of the risk estimates between the screened and unscreened populations, irrespective of the two statistical methods applied (DerSimonian and Laird and likelihood-based). Interestingly, the penetrance estimates were not consistently lower or higher for the screened and unscreened populations across genes and sexes, potentially a result of heterogeneity of included studies.

Risk estimates have changed significantly over the years, with larger sample sizes, inclusion of population-based MMR carriers, and correction for ascertainment bias. Recent studies have been conducted using large, prospective, multinational databases of interventions and outcomes according to the gene mutated such as those from the Prospective Lynch Syndrome Database (PLSD). The first of these studies (outcomes in those without previous cancer) (2) is included in the Wang et al. (1) meta-analysis. These studies have begun to provide some much-needed clarity, particularly with respect to carriers of *MSH6* and *PMS2* mutations. Comparing age-, gene-, and sex-specific lifetime cumulative penetrance estimates to age 70 years for colorectal cancer from the Wang et al. (1) meta-analysis, with results from the PLSD expanded cohort of 6350 MMR carriers (3), it is reassuring to note that there is significant overlap in the 95% confidence intervals, although the specific point estimates vary (Table 1).

**Table 1.** Cumulative cancer penetrance at age 70 years stratified by sex and gene: comparison of results from meta-analysis and analysis of prospective data

Data source	Sex	MLH1	MSH2	MSH6	PMS2
		Cumulative Penetrance % (95% CI)	Cumulative Penetrance % (95% CI)	Cumulative Penetrance % (95% CI)	Cumulative Penetrance % (95% CI)
Meta-analysis: Wang et al. (1)					
M		43.9 (39.6 to 46.6)	54 (49 to 56.3)	12 (2.4 to 24.6)	–
F		37.3 (32.2 to 40.2)	38.6 (34.1 to 42)	12.3 (3.5 to 23.2)	–
Prospective: Domingques-Valentin et al., expanded PLSD (3)					
M		52.8 (45.2 to 61.6)	46.3 (36.9 to 58.8)	11.7 (4.7 to 35.2)	3.4 (0.6 to 34.5)^a^
F		44.1 (37.4 to 51.8)	41.9 (34.9 to 49.7)	20.3 (11.8 to 40.5)	

^a^ Estimates for men and women combined. CI = confidence interval; F = female; M = male; PLSD = Prospective Lynch Syndrome Database.

Regrettably, reliable risk estimates and the place for surveillance in *PMS2*—when to start and how often to repeat—remain most controversial at present. Pal Moller, leader of analytics for the PLSD, has concluded from the PLSD data that the colorectal cancer risk is sufficiently low in *PMS2* as to warrant a markedly relaxed approach to surveillance. Despite this, the National Comprehensive Cancer Network guidelines continue to provide for colonoscopy surveillance that is as aggressive as that for the more highly penetrant *MLH1* and *MSH2*. Still fewer data inform cancer risk in *EPCAM* carriers, not necessarily because the risks are as low as with *PMS2* but because *EPCAM* seems to simply be very infrequently implicated in series of known or suspected hereditary nonpolyposis colorectal cancer.

Much of the effort in the Wang et al. (1) analysis focused on outright exclusions of or statistical corrections for the limitations inherent in studies with flawed ascertainment, redundant data sources, and the like, leaving only a handful of studies suitably lending themselves to meta-analytic aggregation. Even the studies that were included did not always fully disaggregate according to sex and gene that was mutated. Only three were able to provide risk estimates for *MSH6* carriers and, as noted, no attempt could be made to offer *PMS2* or *EPCAM* risks. In addition, the presence and effects of colorectal screening varied between otherwise comparable studies, and thus, such impacts could not be directly measured. As well, it was not always possible to distinguish those with a prior colorectal cancer resection from those who were merely pathogenic variant carriers.

It would behoove those of us who perform genetic testing on, and who clinically manage, patients with MMR pathogenic variants to be sensitive to the limitations of the data aggregations relied on in this meta-analysis. Although the age of onset curves for the highly penetrant genes generally support existing clinical practice guidelines insofar as colon surveillance is concerned, much uncertainty remains as to the risk in *MSH6*, *PMS2*, and *EPCAM* carriers. On one hand, there is pressure to relax surveillance (later initiation, perhaps less frequent exams) based on the supposition of lower age-specific risk in these patients. This is countered by the persistent concern by others that there are insufficient outcomes data, with or without interventions (endoscopic polypectomy), to warrant such a seemingly cavalier approach. This tension will persist despite the best efforts of Wang and colleagues (1). Design of future outcomes studies will need to pay heed to the methodologic pitfalls of post hoc analysis and try to prospectively collect data in a fashion that lends itself to robust analysis.

## Funding

None.

## Note

**Conflicts of interest:** The authors have no conflicts of interest to disclose.

**Role of the author:** Both authors wrote and approved the final version of the manuscript.
